# Supplementary figures and images for: Successful Treatment of an Esophago-Tracheobronchial Fistula Using Double Stenting to Correct Initial Stent Migration: A Case Report and Literature Review
Source: J Clin Med. 2024 Dec 4;13(23):7382. doi: 10.3390/jcm13237382 (PMC11642276; doi:10.3390/jcm13237382)

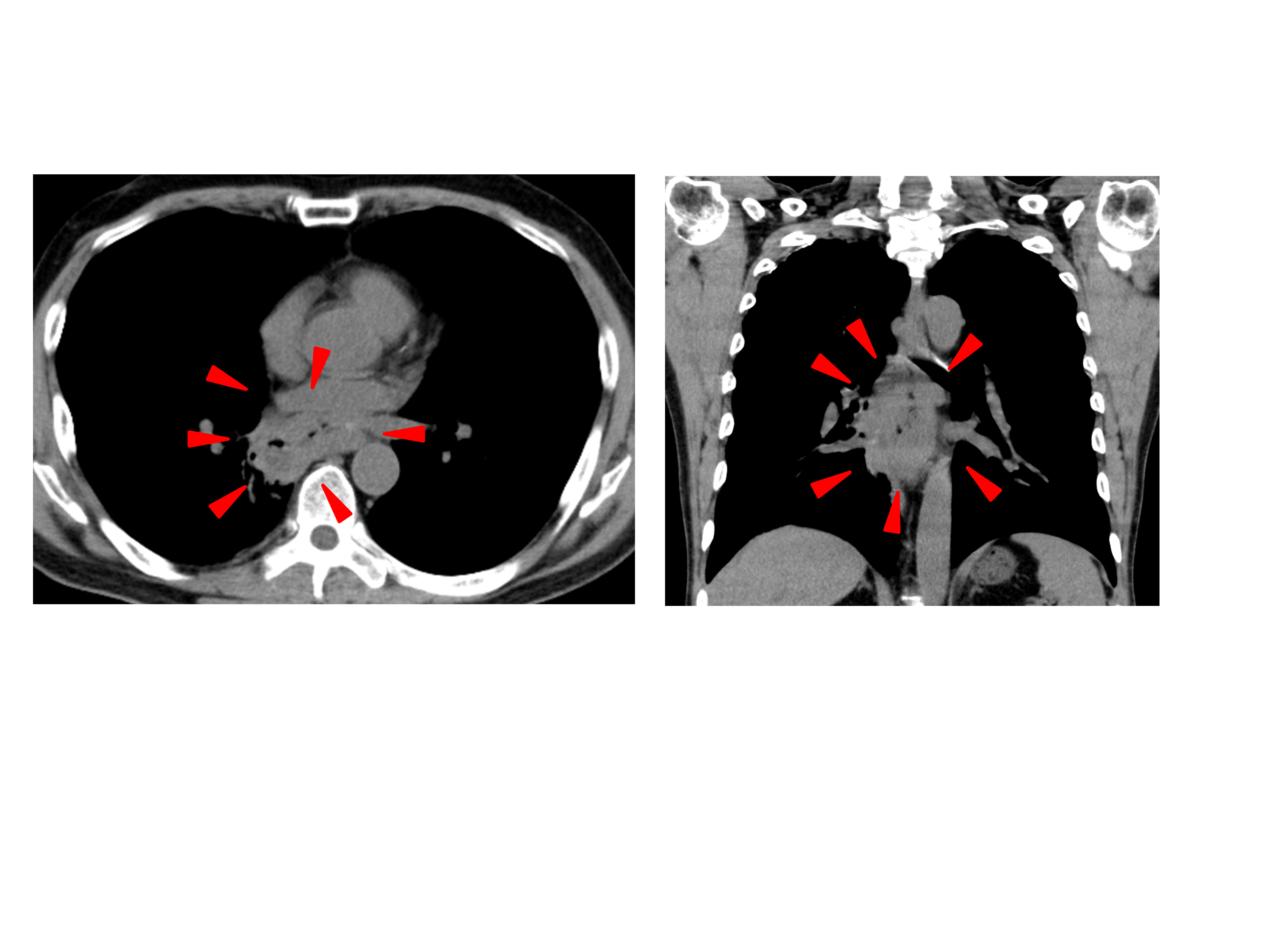

Supplement: Supplementary file 1 [file jcm-13-07382-s001.zip › Suppl. Figure S1.tif]

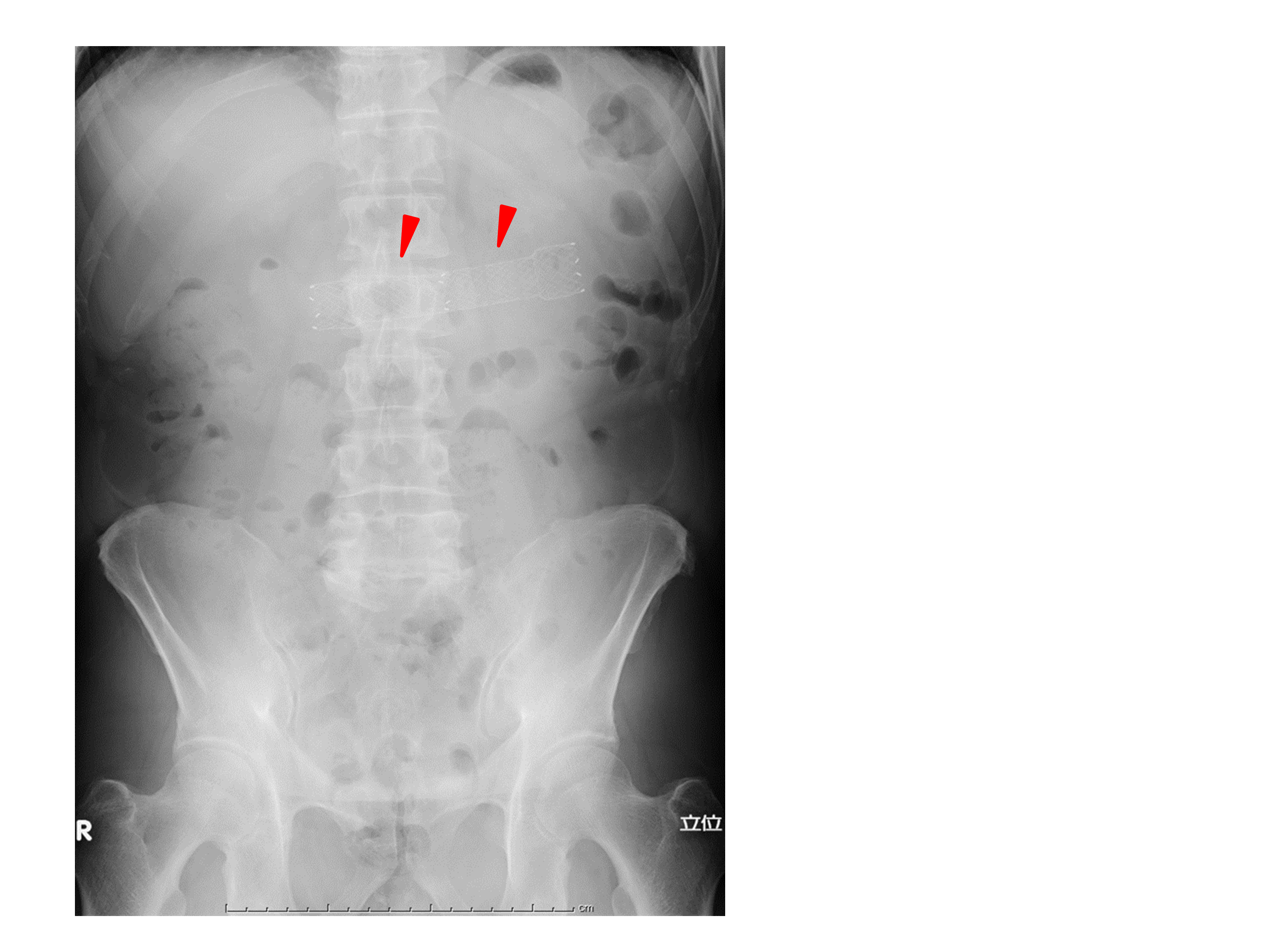

Supplement: Supplementary file 1 [file jcm-13-07382-s001.zip › Suppl. Figure S2.tif]

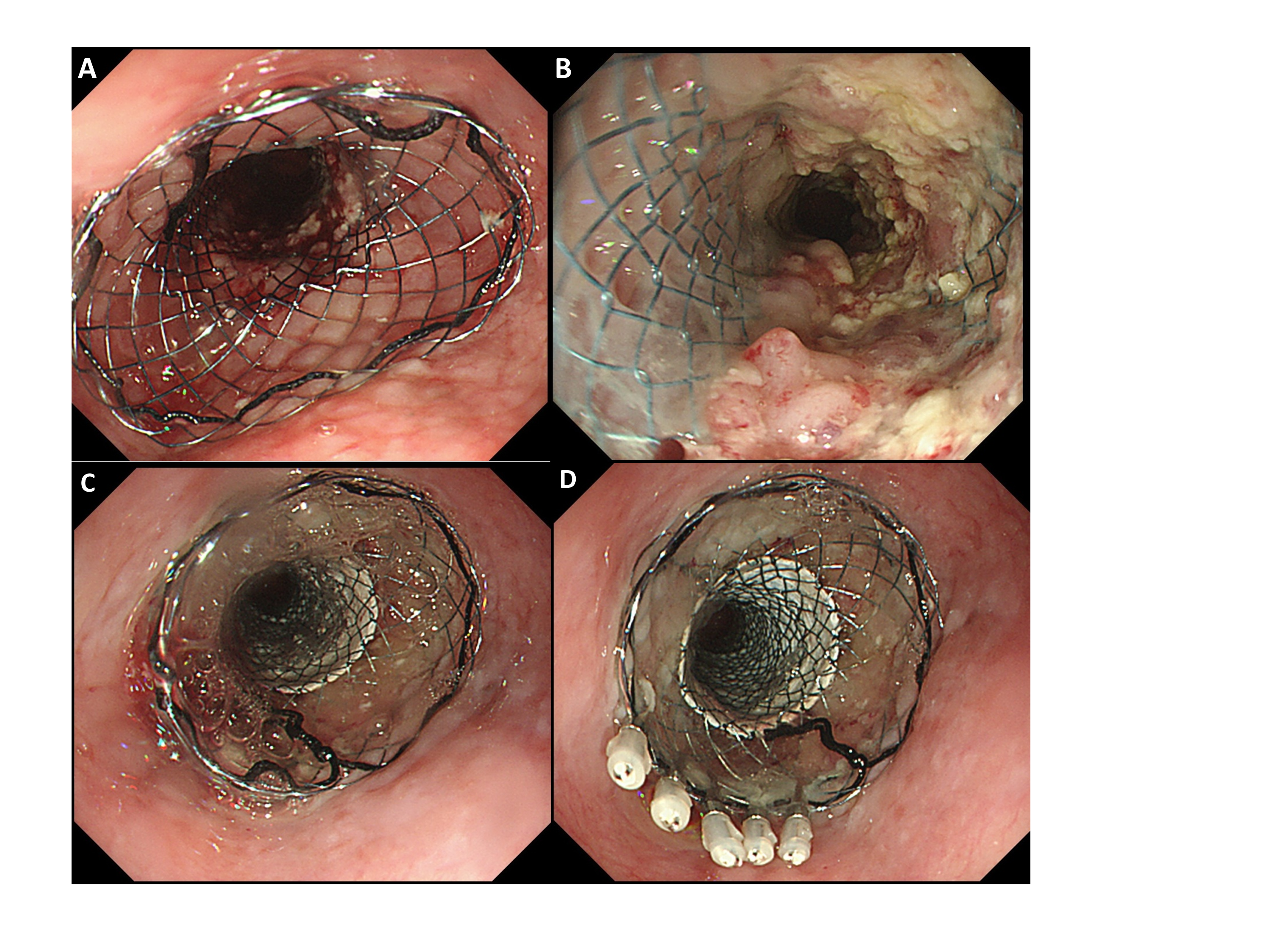

Supplement: Supplementary file 1 [file jcm-13-07382-s001.zip › Suppl. Figure S3.tif]
